# Implementation of pediatric and fetal postmortem imaging into clinical practice: a multi-society statement from the ESPR, SPR, SLARP, AOSPR, WFPI, and IAFR

**DOI:** 10.1007/s00247-026-06639-6

**Published:** 2026-05-23

**Authors:** Aurélie DʼHondt, Stacy Goergen, Elka Miller, Sharon Gould, Susan Shelmerdine, Willemijn Klein, Michael Aertsen, Ajay Taranath, Marta Gomez-Chiari, Teresa Victoria, Padma Rao, Lizbet Perez-Marrero, Manisha Jana, David Perry, Maiko Yoshida, Osamu Miyazaki, Rick R van Rijn, Owen J Arthurs

**Affiliations:** 1 Department of Diagnostic and Interventional Radiology, Hospital for Sick Children, University Avenue, Toronto, 555, ON M5G1X8 Canada; 2 Université Libre de Bruxelles, Brussels, Belgium; 3 Monash Health, Melbourne, Australia; 4 Department of radiology and radiological sciences, Monash University, Melbourne, Australia; 5 Department of medical imaging, University of Toronto, Toronto, Canada; 6 Nemours Childrenʼs Health System, Jacksonville, United States; 7 Thomas Jefferson University, Philadelphia, United States; 8 Department of clinical radiology, Great Ormond Street Hospital, London, United Kingdom; 9 Great Ormond Street Hospital Biomedical Research Centre, London, United Kingdom; 10 Department of medical imaging, Radboud University Nijmegen Medical Centre, Nijmegen, Netherlands; 11 Department of radiology, Universitair Ziekenhuis Leuven, Leuven, Belgium; 12 Translational MRI, Department of Imaging and pathology, KU Leuven, Leuven, Belgium; 13 Womenʼs and Childrenʼs Hospital, Adelaide, Australia; 14 Faculty of health and medical sciences, University of Adelaide, Adelaide, Australia; 15 Diagnostic imaging department, Hospital Sant Joan de Déu Barcelona, Barcelona, Spain; 16 Massachusetts General Hospital, Boston, United States; 17 Royal Childrenʼs Hospital, Melbourne, Australia; 18 Diagnostic imaging department, Clínica Alemana, Santiago, Chile; 19 Department of radiodiagnosis and interventional radiology, All India Institute of Medical Sciences, New Delhi, India; 20 Starship Childrenʼs Health, Auckland, New Zealand; 21 Chiba University Center for Education Research in Legal Medicine, Chiba, Japan; 22 Department of radiology, National Center for Child Health and Development, Tokyo, Japan; 23 Department of radiology and nuclear medecine, Amsterdam University Medical Centers, Amsterdam, Netherlands

**Keywords:** Autopsy, Child, Magnetic resonance imaging, Perinatal death, Postmortem imaging

## Abstract

Postmortem imaging (PMI) following fetal, perinatal, and pediatric demise is increasingly requested and used in clinical practice, either as a non-invasive adjunct or alternative to conventional autopsy. Use of such techniques in clinical departments may face numerous barriers and challenges, varying between centers, countries, and continents, influenced by differences in healthcare systems, funding sources, and local regulations. Furthermore, different issues arise for each specific indication of pediatric PMI (such as intra-uterine demise, termination of pregnancy for confirmation of anatomical abnormalities, and forensic investigations of infants and childhood deaths) since the processes, goals of the investigation, and involved teams vary with indication and/or resource setting. This multi-society position statement describes how different countries currently deal with those challenges, and proposes universal consensus recommendations, enablers, and practical tips to facilitate the successful implementation of fetal and pediatric PMI across medical imaging departments. Implementation to a minimum standard will help resource allocation, collaboration between centers, and multi-center studies in a new field where patient data is scarce.

**Graphical Abstract:**

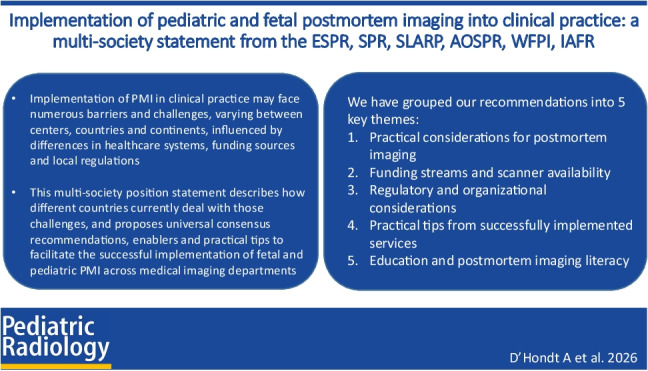

## Introduction

Pediatric postmortem imaging (PMI) is increasingly used, both for the assessment of unexpected deaths and for the evaluation of fetal malformations after termination of pregnancy (TOP) or intra-uterine fetal demise (IUD) [1]. Postmortem radiology started to receive widespread attention with the use of postmortem CT by the Virtopsy group in Zurich [2]. What initially started as targeted imaging for forensic medicine quickly found its way into whole-body clinical radiology and pediatric radiology. Since then, there have been numerous studies by research groups worldwide assessing the value of PMI in addition to or instead of conventional autopsy [3]. During the same period, acceptance rates of invasive fetal and pediatric autopsy examinations have dropped worldwide to only 30–40% [4], despite invasive autopsy known to provide additional useful information in approximately half of all cases [5–7]. In combination with an international shortage of perinatal pathologists and pressure to deliver perinatal autopsy services, this has driven the use of non-invasive imaging. There is now a wide range of proven imaging modalities available, including postmortem conventional radiography (PMCR), postmortem ultrasound (PMUS), postmortem CT (PMCT), postmortem MRI (PMMRI), and micro-CT [8]. There is a growing interest in newer imaging techniques such as photon counting CT, with developments continuing in high-field MRI, addition of intravenous contrast/ventilated imaging, and the use of AI models. The ESPR Postmortem Taskforce has provided consensus expert recommendations on how to perform some of these various imaging tests [9, 10].

However, implementation of such techniques in clinical radiology departments can face numerous barriers and challenges. These obstacles vary between institutions and countries and are influenced by differences in infrastructure, healthcare systems, funding sources, and jurisdictional laws and regulations [11, 12]. Furthermore, different barriers may arise for specific indications for pediatric PMI, which vary from intra-uterine demise, termination of pregnancy because of anatomical abnormalities, sudden unexpected death in infancy (SUDI), and forensic investigation of infant and child deaths. For radiologists and clinicians interested in providing a fetal and/or pediatric postmortem imaging service, where to start and what to do first can be unclear.

This joint multi-society position statement has been drawn up and endorsed by international experts to facilitate the successful implementation of PMI in medical imaging departments. The statement has been endorsed by representatives from multiple international pediatric imaging societies including the:

- European Society of Paediatric Radiology (ESPR),
- Society for Paediatric Radiology (SPR),
- Sociedad Latinoamericana de Radiología Pediátrica (SLARP),
- Asian Oceanic Society of Paediatric Radiology (AOSPR),
- World Federation of Pediatric Imaging (WFPI),
- International Association of Forensic Radiographers (IAFR),

as well as several national radiology societies including:

- Japanese Society of Pediatric Radiology (JSPR) and
- Canadian Society of Pediatric Radiology (canSPR)
- Royal Australian and New Zealand College of Radiologists (RANZCR)

This statement aims to systematically address the challenges and propose agreed recommendations across a number of key themes:

1. Practical considerations for postmortem imaging
2. Funding streams and scanner availability
3. Regulatory and organizational considerations
4. Practical tips from successfully implemented services
5. Education and postmortem imaging literacy

## Practical considerations for postmortem imaging

It is essential to understand the broad range of factors that can influence clinical practice in establishing a PMI service. In this manuscript, PMI refers to two principal clinical contexts that may be encountered within a maternal/children’s hospital setting. The first is fetal PMI, performed following TOP to confirm or refine antenatal diagnoses, or after IUD to investigate potential causes or exclude underlying fetal anomalies. The second is pediatric PMI, encompassing cases from birth to 16 years of age, with a range of indications comparable to those for conventional autopsy in this age group. These include non-forensic (sudden unexpected death in infancy (SUDI) and childhood (SUDC), unexpected in-hospital deaths, accidental traumas) and forensic investigations (known or suspected non-accidental injury, also termed suspected physical abuse and inflicted injury).

In this position statement, our goal is to provide recommendations applicable to both clinical settings, with specific considerations highlighted where relevant.

**Fig. 1:**
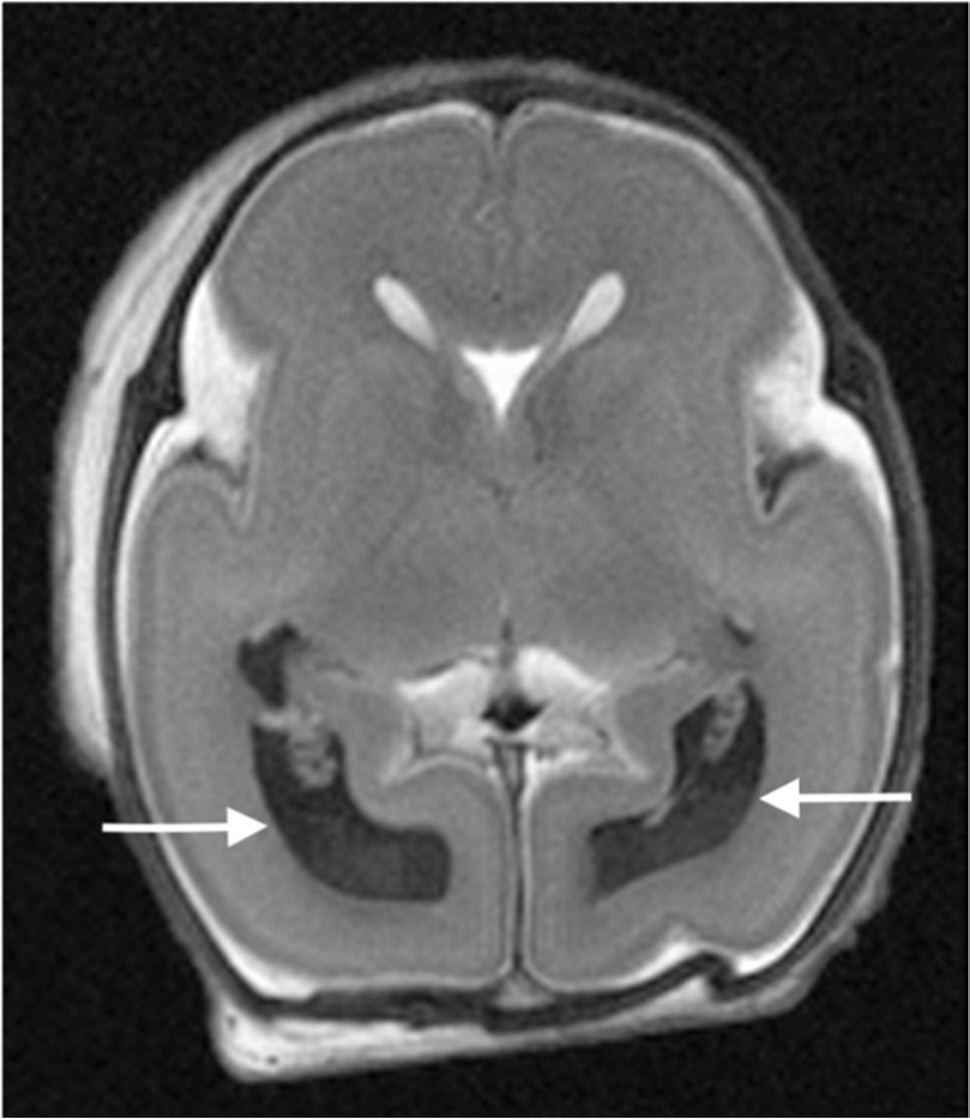
Axial T2-weighted postmortem magnetic resonance image in a 25-week-old female fetus showing classical intraventricular hemorrhage (*arrows*). Adapted with permission [33]

**Fig. 2:**
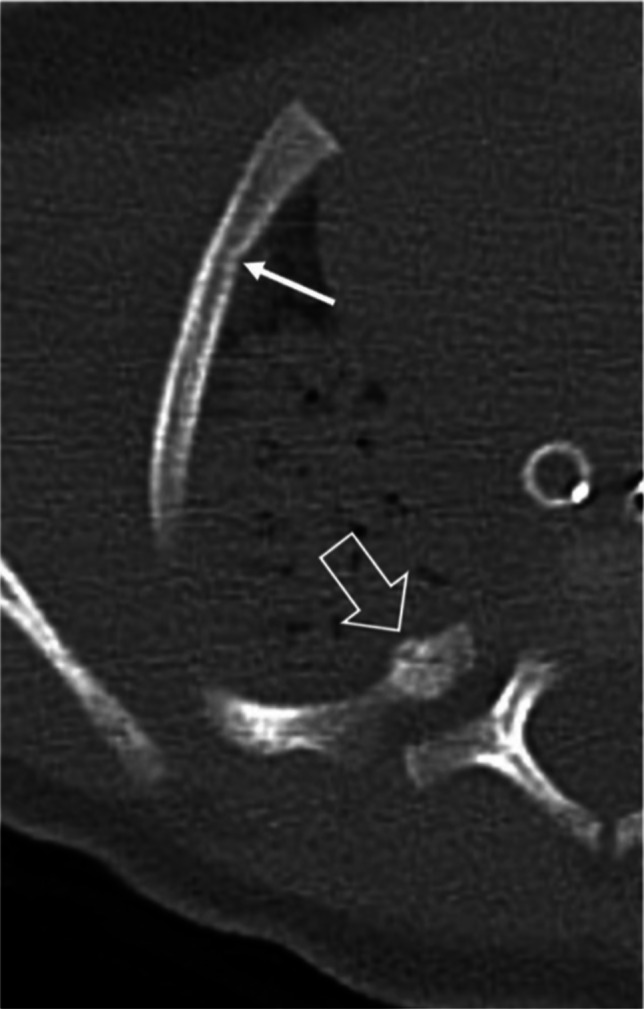
Axial postmortem CT of the chest in a 60-day-old boy following suspected physical abuse. High-dose acquisition and sharp reconstruction kernels allow high-quality imaging demonstrating in this case buckle deformity of the anterior rib (*closed arrow*) likely related to resuscitation, and a healing posterior rib fracture (*open arrow*), consistent with physical abuse. Reproduced with permission [34]

**Fig. 3:**
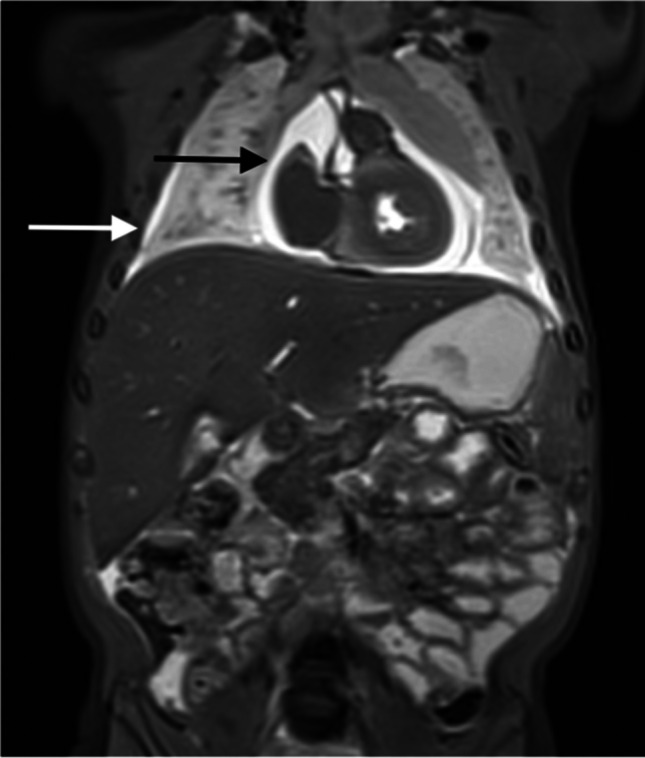
Normal thoracoabdominal postmortem magnetic resonance imaging in a 3-month-old boy. Coronal T2-weighted image shows a small amount of pericardial fluid (*black arrow*) and pleural fluid (*white arrow*), consistent with normal postmortem changes

**Fig. 4:**
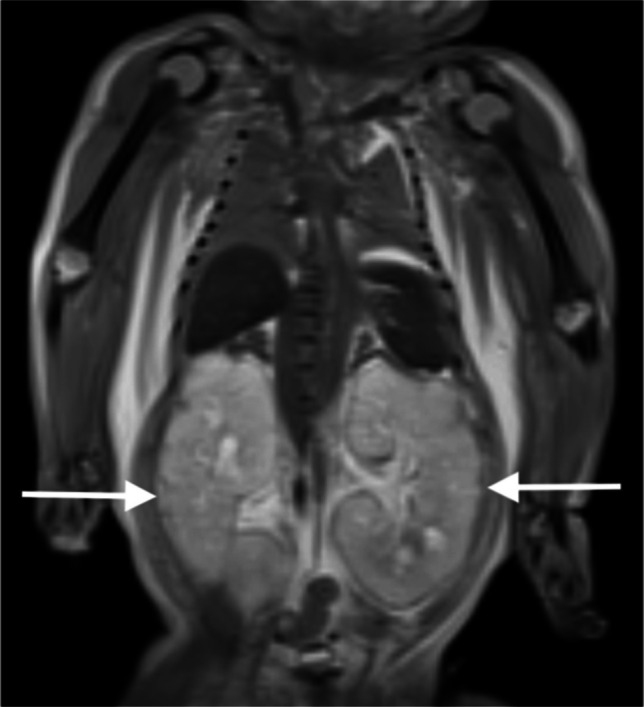
Coronal T2-weighted postmortem magnetic resonance image in a 33-week-old male fetus showing symmetrically enlarged kidneys (*arrows*) in the setting of autosomal recessive polycystic kidney disease. Adapted with permission [33]

Ethical and religious sensitivities should be carefully considered at every stage, as they can strongly influence parental consent and the acceptance of imaging-based alternatives to autopsy. Addressing these foundational considerations helps ensure that PMI is delivered in a respectful, effective, and context-sensitive manner, thereby building trust with families and referrers and enhancing the overall quality of care. As for all imaging requests, clinicians should weigh the likely diagnostic yield of PMI in the context of available information and discuss limitations during the consent process with the referrer, recognizing that this may be a limited resource in line with sustainability principles. Table 1 describes some theoretical prerequisites to consider before starting a PMI service, along with key references for further reading. An overview of performing and reporting PMI can be found in a recent comprehensive imaging guide [32]. Examples of PMI are provided in Figs. 1, 2, 3, and 4.

**Table 1:** Practical considerations for postmortem imaging

	Taskforce guidance
What is the best imaging modality to use?	Depends on referral indication, age of the child (gestation if fetus and size), and availability of local resources and expertise For in-utero death, the optimal imaging modality for fetal and perinatal death has been described [1, 13] For small fetuses weighing less than 500 g (postmortem body weight) or aged less than 18 weeks of gestation, more advanced imaging techniques such as high-field MRI (>7 T) or PM micro-CT are required, although 3 T MRI (and to a lesser extent 1.5 T) may still provide useful information when such equipment is unavailable [14] For mid-second and third trimester perinatal losses (>18 weeks’ gestation or 500 g), PMMRI (preferably 3 T [15]) and PMUS are the most appropriate imaging modalities where available PMCT or postmortem radiographs can identify bony abnormalities, permit long bone measurements to facilitate gestational age estimation, and to confirm intrauterine growth abnormalities
When should PM imaging be performed?	As soon as reasonably possible after delivery or demise, to minimize postmortem changes Recommended within 72 h (and ideally within 24 h whenever possible), with the body stored refrigerated at around 4 °C until imaging can occur Artificial rewarming is not necessary prior to imaging but letting the corpse rewarm by itself up to room temperature does improve the image contrast on PMMRI [16, 17] Freezing of the body is not recommended due to impairment of normal tissue contrast and signal characteristics when tissue is frozen [18, 19]
What clinical information does a radiologist have to know prior to performing a PMI study?	A standardized referral template for perinatal postmortem imaging has been recently established by consensus experts from the ESPR postmortem taskforce and is available for download [20] The required referral information includes: patient’s identification, gestational age, gender, type of demise, date and time of fetal demise, singleton/multiple pregnancy, clinical obstetrical history, prenatal imaging findings, provisional clinical diagnosis, and referring physician’s information For forensic pediatric deaths, clinical information (such as drug history, past medical and social history, and antemortem imaging), along with a copy of the police report, should be included
What imaging protocols should be used in clinical practice?	For PMMRI, a recent expert consensus has proposed standardized protocols for pragmatic clinical use, with the goal of minimizing scanning time to integrate PMI cases into busy clinical MRI workflows [21]. The “minimal” PMMRI clinical protocol includes only essential sequences needed, such as three-dimensional (3-D) isovolumetric T2- and T1-weighted sequences of the brain and chest-abdomen-pelvis. The “ideal” PMMRI protocol includes the full set of sequences including 3-D isovolumetric T2- and T1-weighted sequences of the whole body (the brain, chest-abdomen-pelvis, and limbs) along with further brain imaging For PMCT, a standardized, high–resolution, low-noise imaging protocol has also been established through expert consensus and includes full-body coverage in the axial plane, with reconstructions in coronal and sagittal planes, as well as 3D volume rendering [10] Practical guides on how to perform PMUS and micro-CT have also been developed [22, 23]
How to report PM imaging?	Several publications available on normal findings on postmortem MRI [24], US [25], and CT [26], as well as the imaging changes that typically result from cardiopulmonary resuscitation [27] and maceration [28] Guidance on how to report a postmortem CT (which include a reporting template) has been provided [29] and the Royal Australian and New Zealand College of Radiologists has provided a template for PMMR reporting following perinatal death [30] A suggested template for PMUS in perinatal deaths following a similar structure to the suggested reporting template for antenatal ultrasound as guided by ISUOG is provided in the supplementary material of this reference article [31] More information on how to perform and report PMI is available [32]

*CT* computed tomography, *D* dimensional, *ISUOG* International Society of Ultrasound in Obstetrics and Gynecology, *MRI* magnetic resonance imaging, *PM* postmortem, *PMCT* postmortem computed tomography, *PMI* postmortem imaging, *PMMRI* postmortem magnetic resonance, *PMUS* postmortem ultrasound, *T* tesla, *US* ultrasound

## Funding streams and scanner availability for pediatric postmortem imaging

Despite the decline in parental acceptance for conventional autopsies and the increased use of PMI in clinical settings, evidence suggests that the primary barrier to widespread implementation includes the lack of dedicated funding, incomplete or partial funding, or research grant funding instead of standard clinical imaging remuneration [35–37]. Where cross-sectional PMI has shifted from research to routine clinical practice, mainstream funding for service provision has often not followed.

In a recent survey conducted among members of the ESPR Postmortem Taskforce, only a third of institutions reported receiving full funding or reimbursement for their current medical (non-forensic) PMI practice, whereas forensic postmortem imaging was fully reimbursed in over 50% of institutions [11]. In North America, PMI regardless of indication was reimbursed in less than 15% of institutions surveyed [12], and costs were typically absorbed by the radiology department or the hospital itself; none was derived from medical insurance. The primary sources of funding for PMI were national/state funding streams or prearranged fees per study through coroners or medical examiners.

In order to begin a PMI service, therefore, hospital administrators must first be persuaded to support a program that currently lacks reimbursement, without a clear mechanism for future sustainability, for example through specific billing codes. Most survey respondents believed that national/state funding or an insurance-based stream would be the preferred method for funding postmortem imaging services [11]. The development of national and/or international guidelines for postmortem imaging referral, acquisition, and reporting - initiatives supported by multiple international postmortem task forces - could help standardize practice and strengthen the case for formal funding and reimbursement pathways [9, 38].

Funding for PMI encompasses several key components, including provision imaging equipment and healthcare professionals time, remuneration and transport costs required to move the body to the scanning facility, and remuneration for radiologists to report the studies and to attend multidisciplinary meetings to enable autopsy reports (integrating PMI and autopsy if performed). Additional funding may be required for mortuary space or refrigeration units to facilitate storage. Furthermore, investment in training for both radiographers (*in this document, “radiographers” includes imaging technicians, mortuary technicians, radiological technologists and other professions involved in the acquisition of post mortem imaging*) and radiologists, through dedicated educational courses or conference participation, is essential to ensure high-quality service delivery.

In some countries with more mature workflow (e.g., the Netherlands, Switzerland, UK, Australia, Japan, the USA, and Canada), several pathology departments or forensic medicine facilities are equipped with dedicated MRI and/or CT scanners, and imaging is already integrated as part of a comprehensive postmortem assessment. In such settings, imaging is performed on-site, alleviating pressure on busy hospital clinical scanners (although the current throughput of 10–100 cases/year suggests that this is not a significant factor [38]), and the resulting studies are interpreted separately by radiologists or experienced pathologists. The medical imaging departments then invoice the coroner’s office for the radiology report. Another potential strategy to optimize scanner time and availability for these relatively unpredictable cases is to schedule postmortem imaging before the start or after the end of the routine clinical schedule. Although this approach may incur additional costs due to extended personnel time, e.g., MRI technologists or mortuary staff, it facilitates body transport without interfering with clinical patient flow, addressing both logistical and privacy concerns. Indeed, emotional and psychological considerations must also be acknowledged when scheduling postmortem imaging alongside routine clinical scans. Some patients or their families may feel distressed upon learning that a deceased individual was recently imaged on the same scanner, regardless of whether they witnessed the transfer or were assured of appropriate cleaning protocols.

Given the long imaging waiting lists for both emergent and non-emergent clinical cases, funding for PMI may not be seen as a priority. Health economic impact assessments have yet to be conducted, but they may provide valuable insights into how and where PMI may offer cost-saving opportunities and improve efficiency within pathology and mortuary services. In addition, PMI can provide critical information in cases where parents decline conventional autopsy, supporting both diagnostic clarification and offering closure to aid in the grieving process [39]. The potential cost savings involved in preventing future pregnancy losses if phenotyping through PMI enables a genetic diagnosis to be made remains largely unrecognized and unquantified. This will vary by clinical indication and by the local availability and cost of genetic testing, particularly for single-gene disorders. Key statements to improve funding are summarized in Table 2.

**Table 2:** Postmortem imaging key statements

PMI funding streams key statements	PMI regulatory and organizational key statements	PMI educational key statements
**Establish sustainable funding mechanisms:** Advocate for national or state-level funding dedicated to PMI Work with policymakers to develop and implement billing codes for PMI Encourage public and private insurers to recognize the value of PMI and reimburse where applicable	**Understand and adapt to local legal frameworks:** Identify how medicolegal death investigations are organized nationally or regionally Build relationships with local forensic and perinatal pathologists, and medical examiners to support PMI integration into the sequencing of death investigations Use recommendations from forensic and pediatric radiology societies when advocating for standardized practices Ensure local legal and ethical frameworks are understood and complied with by everyone involved in imaging after pediatric and fetal deaths	**Specialized PMI training:** First “train the trainers” in order that they may bring back useful skills to their department Incorporate PMI into residency and fellowship curricula Create dedicated PMI fellowship programs for pediatric radiologists Develop structured educational courses and certified training modules for both radiologists and radiographers Promote and facilitate observerships and site visits to centers with established PMI expertise
**Conduct health economic assessments:** Support studies to evaluate the cost-effectiveness of PMI and the creation of a meaningful institutional business plan Demonstrate potential time savings and service efficiencies in mortuary and pathology workflows For example, consider the diagnostic yield of PMMRI compared to conventional autopsy after review of prenatal investigations, genetic testing, and placental, cord, and membrane histopathology for unexplained stillbirths	**Create a structured logistical PMI pathway:** Include body transport logistics, preserving the forensic chair if necessary, outlining staff responsibilities, and scan scheduling This will ensure smooth, respectful, and clinically appropriate timing of workflow across departments	**Leverage professional societies:** Promote the use and advantages of PMI Develop multidisciplinary guidelines to standardize and integrate PMI use into the investigation of perinatal and child deaths [40]
**Engage radiology department and hospital administration:** Justify the institutional investment in PMI, highlighting the clinical, ethical, and emotional value Allocate dedicated scanner time, planning for low but variable volume of PMI cases Optimize use of non-clinical scanners where available Work with patient/parental child loss and bereavement charities and local religious groups to vocalize need for PMI service	**Establish multidisciplinary coordination:** Form a core team of pediatric radiologists familiar with PMI and setup shared for all relevant stakeholders (referring clinicians, technologists, radiologists, pathologists, admin staff) to streamline coordination from referral to report delivery Maintain direct communication with the requesting physician at all stages	**Develop patient- and family-oriented material:** Create informative brochures or videos explaining the role and value of PMI and where this fits within the overall investigation of perinatal and child deaths [41] Support clinicians through education, resources, and monitoring of implementation to counsel families about the value of PMI as an alternative and as an adjunct to conventional autopsy

*PMI* postmortem imaging, *PMMRI* postmortem magnetic resonance imaging

## Regulatory and organizational considerations for postmortem imaging

### Regulations

PMI is widely used in Europe and Oceania, with minimal English-language published information on how this is conducted in Africa or Latin/South America. There has been slower uptake in North America, which may partially be related to billing complexities, but more than 50% of the 18 surveyed institutions in a recent SPR survey used PMCT and 24% PMMRI [12]. While similar challenges are encountered globally, medicolegal systems vary significantly between countries and continents, and it is important to understand the specific local regulations surrounding pediatric death investigations, and to establish good communication with local Departments of Justice, coroners and medical examiners, and pathologists, to ensure effective collaboration with medical imaging departments.

#### The USA

##### Medicolegal death investigations

Harty M. et al. have described the specific challenges involved in establishing a pediatric forensic PMI service in the USA [42]. Due to the decentralized nature of medicolegal death investigations, authority over the organization and operation of forensic services falls to individual states and counties. This has resulted in a patchwork of coroner and medical examiner (ME) offices, each functioning under different statutes and with varying levels of funding. In the USA, medicolegal death investigations are conducted by either MEs or coroners, depending on the state and sometimes even by the county or district within each state. Medical examiners are almost exclusively physicians trained in forensic pathology, though the number of trained forensic pathologists in the country is limited. Local laws and political considerations often influence who is responsible for death investigations, and stakeholders may have motivations to maintain the status quo. The lack of a centralized national medicolegal death investigation system makes it challenging to establish a unified forensic imaging service that can coordinate effectively at the state or federal level. Both the National Association of Medical Examiners and the College of American Pathologists have acknowledged the need for standardized forensic practices in the USA, as well as the importance of expanding the role of PMI in routine investigations [43, 44].

##### Fetal death investigations

A significant barrier for fetal PMI in the USA is the widespread use of dilatation and evacuation (D&E) technique for terminations of pregnancies. It often results in fetal fragmentation, making it unsuitable for PMI.

#### Australia

##### Medicolegal death investigations

The Australian coronial system investigates unnatural, unexpected, or unexplained deaths, and comprises coroners and forensic medicine institutes, courts, and the National Coronial Information System (which also collects data for New Zealand). State and territory governments control their own coronial systems. Access to imaging equipment varies by jurisdiction with some having in-house CT scanners and radiography, while others are reliant on local hospitals providing these services. Similarly, there is variation in who interprets and reports on CT scanning performed for forensic purposes in the pediatric population in Australia. MRI is generally unavailable in forensic medical facilities but has recently been installed at the Victorian Institute of Forensic Medicine (VIFM; Melbourne; June 2025); however, children referred to the coroner may have PMMRI performed on a case-by-case basis at the hospital that has cared for them during life, dependent on the availability of technologists, radiologists, and equipment to provide this service. In-hospital postmortem imaging for deaths referred to the coroner is not currently reimbursed by the coronial system.

##### Clinical fetal/perinatal deaths investigations

The large majority of fetal cases are spontaneous or iatrogenic fetal demise (stillbirth) following medical termination of pregnancy for fetal anomalies. Death of neonates and children in a non-forensic context is not only much rarer than stillbirth but is far less likely to be due to uncertain cause or associated with a need to confirm prenatally suspected conditions. Hence, these deaths are less likely to be referred for postmortem imaging than are stillbirths. There is currently no funding through the national insurer Medicare for these services which are provided in state-funded public hospitals with hospital radiology departments usually absorbing the cost of the service; state-based perinatal postmortem services may provide variable reimbursement to hospital radiology departments for MRI and radiography but this remains ad hoc and dependent on locally negotiated arrangements. Postmortem MRI and radiography are the most commonly used imaging modalities, supervised, and reported by local pediatric radiologists. At present, at least one hospital in every state and territory provides these services and in the larger states (>5M population) more than one hospital does so. The Australian Government funded an initiative in 2022 - 2023 to upskill radiologists in perinatal PMMRI and this has resulted in improved access for families and perinatal pathologists despite lack of a dedicated funding stream for these procedures.

#### New Zealand

##### Medicolegal death investigations

As in Australia, any sudden death in Aotearoa New Zealand that is unexpected, violent, or suspicious will be investigated by a coroner. Forensic pathology services are located in four major centers which are geographically distributed along the country, with varying degrees of capacity for forensic investigation. In-house CT is available at the largest centers with connected public hospital radiology departments providing imaging for others. The coroner determines whether postmortem investigation is required, but there is no national policy regarding postmortem imaging investigation in children. Some centers routinely use PMCT and radiographs for all coronial cases while in others the forensic pathologist determines which investigations will be used on a case-by-case basis, in conjunction with public hospital radiology services. In cases where families do not want autopsies, the coroner may decide on less invasive autopsy, typically deciding whether to proceed to internal examination following a PMCT. PMMRI is not known to be performed in coronial cases. Reporting is usually performed by local pediatric radiologists with some reports being outsourced to larger pediatric departments in other centers.

##### Clinical fetal/perinatal deaths investigations

Despite the presence of a single publicly funded health care system and authority (Health New Zealand - Te Whatu Ora), there is wide variability in pediatric postmortem imaging practice across the four health regions and 40 public hospitals of Aotearoa New Zealand. While radiographs and CT are available at all public hospitals, utilization is variable and influenced by local referrer and radiologist preference with no national directive to provide the service. PMMRI, which is almost exclusively used in perinatal deaths, is only available in the largest centers with limited numbers of reporting radiologists located at tertiary/quaternary pediatric centers. All pediatric postmortem imaging is broadly funded within the public health system, with nominal payments between services which partially address additional costs.

#### Canada

##### Medicolegal death investigations

In Canada, medicolegal death investigations are typically conducted by coroners or medical examiners, depending on the province or territory. Autopsies and related investigations are performed in forensic pathology units, often affiliated with hospitals, universities, or provincial forensic science centers. These units handle sudden or unexplained deaths, violent deaths, deaths in custody or institutional care, and pediatric or perinatal deaths if deemed suspicious or unexplained. In some forensic units, imaging suites are integrated into medicolegal services: most commonly with X-ray and CT, though MRI is gradually being introduced. In pediatric cases, imaging studies are typically interpreted by pediatric radiologists within the corresponding province. The medical imaging department invoices the coroner’s office for these services, with costs covered through public funding through the provincial or territorial Ministry of Justice or equivalent agencies (e.g., the Office of the Chief Coroner or the Office of the Chief Medical Examiner).

##### Fetal death investigations

Fetal PMI is gradually being adopted across Canadian institutions, with some centers actively developing protocols and training programs to integrate this modality into perinatal pathology services. However, availability and implementation vary significantly by province and institution. Most perinatal deaths or terminations of pregnancy (TOP) occur in adult maternity hospitals, which poses logistical challenges when transferring cases to specialized pediatric centers. While pediatric radiologists with expertise in PMI can help facilitate coordination, there are no dedicated billing codes. Establishing such codes would require departmental leadership to engage with provincial health ministries, a process that is typically lengthy and complex. Existing wait times for clinical MRI and CT imaging reduce the capacity and willingness to accommodate non-urgent postmortem studies. When PM imaging is part of a research clinical project, funding is often sourced from hospital departments, academic grants, or institutional resources.

#### Latin/South America

PMI in Latin America is still new and faces numerous challenges. There are significant disparities in resources among public, private, and university hospitals, and access to advanced imaging technologies varies widely across countries in the region. In addition to logistical barriers, cultural and legal factors also hinder the adoption and integration of PMI in some countries. In 2023, an unpublished survey on the use of PMI in Latin America led by Perez-Marrero provided valuable insights into regional trends and obstacles, from 99 pediatric radiologists from nine Latin American countries (primarily from Chile, Argentina, Brazil, and Peru). While awareness of PMI was high, nearly two-thirds did not use it in their clinical practice. Among the small group of professionals actively using PMI, applications were diverse. PMI was employed as an alternative or adjunct to conventional autopsy, particularly in perinatal and forensic contexts, and some respondents reported its use in specialized fields such as paleontology and embryology. One private institution in Chile implemented a minimally invasive autopsy program for unexplained perinatal deaths approximately 5 years ago. That program includes the use of PMMRI and has been presented at regional radiology conferences, generating growing interest in PMI among pediatric radiologists. Similar initiatives exist in select institutions in Brazil and Argentina, although these are not limited exclusively to pediatric cases. Further professional networking is required to advance the field and improve access to PMI across the region.

#### Asia

##### India fetal death investigations

In India, maternal and infant health-related imaging investigations are state-sponsored under the *Janani Shishu Suraksha Karyakram* (JSSK). As a result, fetal PMI following termination of pregnancy can be performed without billing concerns.

##### Medicolegal death investigations

However, the cost of pediatric PMI in older children is typically borne by the parents, which serves as a significant deterrent to its widespread use. While imaging performed in government institutions can be exempted from payment, i.e., the cost may be covered by the institution at the clinician’s discretion; there are currently no established billing guidelines for this specific scenario in most parts of the country.

##### Japan: medicolegal death investigations

The Japanese death investigation system has historically been controlled by the police organization, focusing on criminal cases, and the forensic autopsy rate is extremely low (<2% for all deaths). The medical examiner system was introduced in the 1940 s but only adopted in certain areas. In 2012 following new death investigation laws which formally included the use of postmortem imaging, the police began contracting with hospitals to perform postmortem CT (PMCT) scans for cases under investigation [45], although uptake remains variable. As of 2022, approximately half the forensic medicine facilities in Japan have introduced CT scanners to facilitate determination of the cause of death, including all infantile and childhood deaths. The Japanese Ministry of Health, Labor, and Welfare convened a committee on the implementation of postmortem imaging in 2009, which recommended that postmortem imaging should be performed in all cases of pediatric death. As a result, although there are no accurate statistics on a national scale, postmortem imaging is no longer uncommon in large facilities that handle pediatric emergency medical care. Postmortem images are interpreted by forensic pathologists or radiologists affiliated with the facility; however, facilities with radiologists with a subspecialty in the interpretation of postmortem images are limited (for example to the University of Tokyo, Chiba University, and Fukui University). To overcome these existing challenges, the forensic radiologists group set up a working group to conduct a workshop for interpretation of postmortem images in 2025, and the Japan Radiological Society has also held annual interactive seminars and included postmortem imaging as a symposium topic. Since clinical practices are operated individually at each facility, it is currently difficult to grasp the overall picture, and comprehensive surveys in the future are desirable.

#### Western Europe

While facilities, familiarity, and experience vary with institution and jurisdiction across Western Europe, fetal and pediatric PMI falls into the death investigation approaches which mirror Australia and Canada, with a division into medical (parental consent for non-suspicious deaths) and forensic or legal cases of suspicious and non-suspicious unexplained deaths (for which parental consent may not be required or sought). Within those systems, there remains considerable variation in the use of postmortem imaging to address specific clinical questions.

In the UK, His Majesty’s Coroners are appointed to investigate suspicious deaths in local authorities, and many are willing to employ postmortem CT in forensic childhood deaths to evaluate primarily for evidence of trauma, and postmortem MRI for late fetal or neonatal deaths in families where there is strong religious objection to an invasive autopsy. There are few dedicated forensic institutes so most pediatric imaging is performed in hospitals, with the advantages of hospital infrastructure, personnel, and live children’s imaging experience, but the demands of children’s imaging services restrict access to those same resources. Several UK institutes are providing late fetal or neonatal death imaging on conventional medical scanners, although reimbursement from the coroner or government remains poorly defined at best, and is in the process of being clarified nationally through service commissioning. Several centers still routinely use plain radiographs despite their relatively low yield for significant abnormalities.

In the Netherlands, there is no mandatory postmortem evaluation in case of natural cause of death. For fetal demise imaging, mostly PMMRI and in case of suspected skeletal dysplasia, conventional radiography will be performed in local hospitals with either local review or review by an experienced pediatric radiologist in the region. There is a voluntary postmortem evaluation of sudden unexpected death in infants and children (PESUDIC) procedure consisting of a step-wise approach led by a pediatrician in collaboration with a forensic physician. For each step in the procedure, parental consent is required. A routine part offered in the PESUDIC procedure is PMCT or PMMR, which is performed in over 90% of cases; imaging contributes to the diagnosis in approximately 40% of cases and in 15% gives the cause of death [46]. For forensic PMI, there are a select number of radiology departments and pediatric radiologists available, who are trained in forensic imaging and have knowledge of the legal system.

In Belgium and in France, there are no billing codes or state funding for PMI, whether fetal or forensic. In fetal cases, examinations are typically registered under the mother’s name. The costs are either absorbed by the department or hospital, or the imaging is performed in a research setting at centers that offer such services.

The key differences in availability, funding mechanisms, and organizational frameworks across the above world regions are summarized in Table 3.

**Table 3:** Regional models for the implementation of pediatric and fetal postmortem imaging

Region/country	Availability	Funding	Framework
USA	Variable pediatric forensic PMI availability; fetal/perinatal PMI limited	No unified system. Billing complexities appear to be an important barrier to wider implementation	Decentralized coroner/medical examiner system with marked state- and county-level variation
Australia	Pediatric forensic PMI is variably available; fetal/perinatal PMI well established (at least in one hospital in each state and territory)	No dedicated Medicare funding; hospital or departments often absorb costs; coronial referrals not reimbursed for in-hospital imaging	State- and territory-based coronial systems; access to imaging equipment and reporting expertise vary by jurisdiction
New Zealand	Pediatric forensic and fetal/perinatal PMI available, but practice is variable across centers; mainly confined to larger hospitals	Publicly funded	Coroner-led system; marked regional variability
Canada	Pediatric forensic PMI available in forensic units or hospitals with variability between provinces; fetal/perinatal PMI is gradually being adopted in some hospitals	Pediatric forensic imaging publicly funded through coronial/justice systems; fetal PMI lacks dedicated billing codes and may rely on departmental or research funding	Coroner-led medicolegal system for forensic PMI; forensic units often linked to universities. Fetal PMI frameworks vary between centers
South America	PMI remains limited, with selected emerging programs in some countries such as Argentina, Brazil, and Chile	No unified system. Some programs are financed by institutions, others are private or paid by the families	Heterogeneous legal, cultural, and logistical contexts across countries. Implementation remains heterogenous at an early stage
India	Pediatric forensic PMI limited; fetal/perinatal PMI accessible	Pediatric PMI often paid by families; fetal PMI state-funded	Limited standardization of PMI pathways
Japan	Pediatric forensic and fetal/perinatal PMI are available in most forensic units and in selected clinical units	Hospitals may be contracted by police to perform PMCT; broader reimbursement structure not clearly defined	Forensic investigation system is police-led and PMI is formally incorporated into death investigation law; uptake remains facility dependent
UK	Pediatric forensic and fetal/perinatal PMI is available in selected centers	Reimbursement from coroner or within National Health System remains incompletely defined but is being clarified nationally	Coroner-based system; most imaging performed in hospitals rather than dedicated forensic institutes; marked regional variability
Netherlands	Pediatric forensic and fetal PMI are both available within structured consent-based pathways	Government-funded for forensic and SUDI cases	For forensic PMI, there are a selected number of radiology departments and pediatric radiologist available. Voluntary stepwise evaluation with parental consent for SUDI cases
Belgium/France	Pediatric forensic and fetal PMI are available in selected centers, but access is variable	No dedicated billing codes nor state funding; costs often absorbed by department/hospitals or supported through research grant; fetal studies often registered under the mother’s name	Service organization depends on local institutional arrangements

Summarized are key differences in availability, funding mechanisms, and organizational frameworks across selected world regions, based on the narrative review

*PMI* postmortem imaging, *PMCT* postmortem computed tomography, *SUDI* sudden unexpected death of infancy

### Organizational considerations

Before the first PMI case is conducted in the imaging department, a clear and detailed protocol must be established. This should cover the transport of the body to the scanner, including a designated route through the hospital (ideally separated from outpatient areas), identification of an appropriate transport box, assignment of responsible personnel, and adequate scheduling. Ideally, the body should be transported by medical personnel or a mortuary technician (in medical cases) or the investigator/police officer (in forensic cases). In forensic cases, this person should also remain with the body in the imaging department throughout the examination in order to guard the chain of evidence. Radiographers should be trained in PMI not only on how it should be done, but even more so on the rationale behind PMI. Discussions need to include all relevant stakeholders, including referring clinicians, technologists, radiologists, pathologists, and administrative assistants, to allow for effective organizational and logistical communication. In each department, a dedicated group of trained imaging professionals who are or used to dealing with PMI should be established to manage all related cases. Current recommendations for PMCT suggest scanning should be performed without removing medical devices (especially in legal cases) [47], although some metalwork must be removed prior to MRI.

Imaging results should be communicated directly with the referring clinician and/or the pathologist who is performing the limited or conventional autopsy, via the established reporting pathway. Establishing an expected turnaround time for the radiology report is crucial, especially when only a small number of radiologists are designated to read these studies, or during weekends and holiday periods. This is particularly important if an autopsy is to be performed, and in the cases of faith deaths where a rapid burial may be sought. If the PMI is not reported prior to the autopsy, valuable information might be lacking that could direct the procedure, target biopsies, and limit the necessary scope of the autopsy. Direct communication with the requesting physician is essential throughout the process, from the initial request to discussing the final results. Key statements around regulation and organization of PMI are summarized in Table 2.

## Practical tips from experts who have successfully implemented postmortem imaging services

### Radiographer engagement and support

Motivating and supporting radiographers is key to successful implementation of a PMI service. The foundation for training and collaborative success lies in a structured training program that includes both theoretical components delivered through formal courses and practical training delivered by experienced, specialized radiographers. This improves both engagement and confidence while safeguarding image quality and forensic integrity [48]. Radiographers on the front line in PMI face emotional and logistical challenges: receiving referrals, coordinating timing which may be outside routine working hours, and having direct contact with the deceased. Comprehensive postgraduate training should highlight the clinical and forensic significance of PMI, ensuring radiographers understand how meticulous technique impacts diagnostic accuracy and the evidential value of images. Those who are comfortable with PMI may be suitable to act as a lead radiographer, while respectfully excusing those who may not feel at ease [48]. Psychological support should be incorporated into local policy. To streamline workflow and acceptability, preparation of the deceased for PMI could include requesting that the mortuary or labor ward prepare the deceased appropriately (e.g., undressed except for a diaper, wrapped in a sheet/swaddle without metallic material).

As with all multidisciplinary teams, radiographers should work closely with radiologists and pathologists to better understand their role and the contribution of PMI in providing answers for grieving families while expanding their own professional knowledge.

### Using a research-to-clinical approach

Many successful programs have begun by integrating PMI within a research framework. This approach allows institutions to develop protocols, demonstrate value, and gather evidence prior to clinical rollout.

### Promoting PMI service

Promotion should occur at multiple levels:

- Internally: Discuss the service with key referrers such as obstetricians, neonatologists, geneticists, and pathologists at their regular clinical meetings. In many institutions, it is now the clinicians who approach radiology departments to initiate PMI services. Once they see how we can help, there is no going back - convincing stakeholders is often straightforward in our experience. It may be necessary to identify gaps in the service that postmortem imaging could solve, such as the high number of fetal autopsies which are non-diagnostic. Radiologists may need to lead on creating written policies and procedures for “how to” refer a patient for PMI, hours of operation, and other logistic issues and continually “training the trainers” in other disciplines about the need to educate new staff on the policies will facilitate smooth running of the process, using international guidelines for support.
- Locally, nationally, and internationally: Share your experience and outcomes at professional meetings, conferences, and congresses to foster broader acceptance and collaboration.

### Engage the pathology department

Initiate discussions with the hospital’s pathology department, which must be on board. Emphasize the collaborative nature of the program and its benefits to clinical teams, grieving families, and both imaging and pathology departments. It can be helpful to incorporate imaging into the existing hospital-approved autopsy consent form. Increasing radiologist access to the pathology department/mortuary as well as active communication between radiologists and pathologists for each case (radiologic-pathologic correlation) via direct discussion or joint rounds. We stress the importance of discussing the imaging findings with the pathologist, if an autopsy is to take place, to ensure that any abnormality seen on imaging may be targeted for biopsy and histopathology. The radiology report is the most important vehicle to support this communication so that anyone performing the autopsy is aware of the imaging findings. This again emphasizes the critical nature of timely reporting as soon as possible after completion of the imaging.

### Flexible scheduling

Allow scans to be performed outside regular clinical hours or empower technologists to select optimal scan times based on their availability. This flexibility reduces stress and improves workflow integration.

### Use of imaging equipment in pathology/forensic departments

Having imaging equipment located directly in pathology or forensic departments streamlines workflow and reduces logistical challenges, though this may not be feasible for all institutions.

### Communication, communication, communication!

Establish mailing groups including all key personnel (referring clinicians, technologists, radiologists, pathologists, and administrative staff) to enable real-time coordination and ensure an efficient, well-organized process for each case.

## Education and postmortem imaging literacy

Currently, there is no formal pediatric postmortem imaging fellowship training nor subspecialty training for qualified radiologists who wish to expand their skills and services to include PMI, although some dedicated courses do exist. Most radiologists performing PMI have self-taught skills by adapting them from clinical pediatric and/or intrauterine fetal imaging experiences, or via postmortem imaging research before starting a clinical service; similarly, many imaging technologists have sought similar peer training or postgraduate courses [20]. Several postmortem imaging courses are available but are predominantly based on adult practices, with variable relevance to dedicated modern pediatric practices.

The ESPR, the SPR, and the International Society of Forensic Radiology and Imaging (ISFRI) all have dedicated pediatric postmortem imaging subcommittees and can provide guidance on training opportunities and may facilitate informal visits with specialist centers that routinely perform PMI. The SPR, ESPR, IAFR, and AOSPR have also organized several courses dedicated to PMI [49–51], and organize regular online meetings discussing anonymized PMI cases, thereby sharing knowledge and experience. The UK Royal College of Radiologists’ training curriculum now includes postmortem imaging as an emerging technique, and the Faculty of Post Mortem Imaging of the Royal College of Pathologists Australia (RCPA) is developing a curriculum for a Fellowship in Post Mortem Imaging [52, 53].

There is a clear need for dedicated training programs – such as structured educational courses and a subspecialty field within pediatric radiology fellowships - for both radiologists and technologists, particularly if more centers are to be encouraged to provide pediatric postmortem imaging services. Educational initiatives outside of healthcare staff could consider creating informative resources for patients and their families as well as clinicians (obstetricians, midwives, neonatologists, perinatal and forensic pathologists) who care for families who have experienced a fetal or child death. Key statements around PMI education are summarized in Table 2.

## Conclusion

Fetal and pediatric postmortem imaging plays a valuable role for families and clinicians following the death of a fetus or child by providing an alternative or adjunct to conventional autopsy in the investigation of their child’s death as well as answers to the most important question of all: why has this happened? To improve and sustain high-quality service provision, it is essential to formalize jurisdictional referral pathways, establish consistent funding mechanisms, and develop dedicated educational resources. Achieving this will require active advocacy from radiologists, including support from international professional societies, and pathologists, in collaboration with policymakers, bereaved parent advocacy organizations, and funding bodies. We hope that our work facilitates the implementation and utilization of pediatric PMI services globally, in clinical and medicolegal settings.

## Data Availability

No datasets were generated or analysed during the current study.
